# Dynamic changes in the transcriptome of tropical region-originated king grasses in response to cold stress

**DOI:** 10.3389/fpls.2025.1511466

**Published:** 2025-02-18

**Authors:** Xianjun Lai, Junfeng Yan, Zihan Chen, Yizheng Zhang, Fan Luo, Guangze Cai, Lang Yan

**Affiliations:** ^1^ Panxi Crops Research and Utilization Key Laboratory of Sichuan Province, College of Agriculture Science, Xichang University, Liangshan, China; ^2^ Chengdu Ke’an Technology Co., Ltd., Chengdu, China; ^3^ Sichuan Key Laboratory of Molecular Biology and Biotechnology, College of Life Sciences, Sichuan University, Chengdu, China; ^4^ Mianyang Youxian Innovation Technology and Industrial Technology Research Institute, Mianyang, China

**Keywords:** king grass, time-course RNA-seq, differential gene expression, gene regulatory network, cold treatment

## Abstract

**Introduction:**

Cold acclimatization in tropical region-originated plants involves complex gene expression reprogramming to adapt to fluctuating temperatures. However, the molecular mechanisms and gene networks regulating cold tolerance in king grass remain largely unknown.

**Methods:**

To address this, we established a full-length reference transcriptome of king grass to enhance assembly quality and performed multiple time-point transcriptomic analyses following cold treatment at 4°C. Differentially expressed genes (DEGs) and transcription factors (TFs) involved in cold stress response were identified and analyzed through clustering and co-expression network analysis.

**Results:**

A total of 13,056 DEGs were identified and classified into nine clusters via k-means analysis. The cold response exhibited three distinct phases: early (before 3 h), middle (6–24 h), and late (48–72 h). Early-responsive genes were enriched in glycolipid metabolism and photosynthesis, middle-stage genes in carbohydrate metabolism, and late-stage genes in cold stress, osmotic stress, and endogenous stimuli responses. Key regulators of the ICE-CBF-COR signaling module, including 13 positive and negative regulators, were identified. The co-expression network further revealed mutual regulatory interactions within this module, highlighting its role in cold stress adaptation.

**Discussion:**

Our findings provide insights into the cold tolerance mechanisms of king grass, offering a genetic basis for modifying cold stress regulators. This research contributes to the broader understanding of low-temperature adaptive mechanisms in tropical plants and supports future breeding strategies for improved cold tolerance.

## Introduction

Cold stress is a major environmental stress factor that adversely impacts on plant growth, development, productivity, and geographical distribution (Zhu, 2016). Cold stress is divided into chilling stress (0–15°C) and freezing stress (< 0°C). Chilling stress adversely affects reactive oxygen species (ROS) homeostasis and energy metabolism in plants, while freezing stress results in cell membrane lesions and structural damage because of the formation of intercellular ice (Chinnusamy et al., 2007; Ruelland et al., 2009). Plants originating from temperate areas have a high chilling stress resistance and can improve their freezing stress tolerance, while those originating from tropical or subtropical regions are susceptible to cold stress and lack of cold acclimation mechanisms, limiting their introduction and growth from south to north China. However, some plants, such as wheat (*Triticum aestivum* L.) and barley (*Hordeum vulgare* L.), have evolved cold acclimation mechanisms at cellular and molecular levels, improving their tolerance to cold stress after exposure to non-freezing temperatures (Guy, 1990; Liu et al., 2022; Thomashow, 1999). Reprogramming gene expression is an adaptive molecular mechanisms in plants responding to cold stress (Liu et al., 2022). In particular, activating the expression of key cold-responsive genes, such as C-repeat/DERB binding factors (CBFs) genes and cold-regulated (COR) genes, can enhance cold tolerance. When plants are exposed to low temperatures, CBF genes are rapidly induced by a set of transcription factors (TFs), such as ICE1 and CAMTA, and the resulting expressed proteins activate the downstream COR genes (Jia et al., 2016; Liu et al., 2018; Zhao et al., 2016). The COR genes are important elements that protect plants from cold damage by encoding osmolyte and cryoprotective proteins and inducing their expression (Kidokoro et al., 2017).

Although transcriptional patterns during cold stress have already been intensively studied in various plant species (Liu et al., 2022; Luo et al., 2022; Park et al., 2021; Thomas et al., 2020), studies on the transient changes and gene networks involved in the regulation of cold tolerance acclimated in tropical region-originated plants are quite limited. As a Pennisetum C4 grass forages, king grass (*Pennisetum purpureum* Schumacher × *P. americanum*), also known as hybrid giant Napier grass, has a high biomass and nutritional value but a low cold tolerance. It is barely distributed in the tropical and subtropical regions worldwide (Fan et al., 2012; Zhao et al., 2019). It was introduced and cultivated in the mountainous areas of southwest China in the 1990s and has been domesticated to adapt to the local habitat. Even so, high-yielding king grass cultivars grow in narrow climatic niches and are known to be less productive at an altitude of >1500 m (Wang et al., 2016). Expanding the growing range of high-yielding king grass has been proposed to achieve economic forage production. Since low temperature has limited the spread of king grass northwards, we were interested in investigating the key genes responsible for enabling its resistance to cold stress. Cold acclimation helps plants cope with cold stress by optimizing antioxidant enzyme activities, osmotic adjustment potentials, and photochemical efficiency. These changes are partially caused by reprogramming gene expression and metabolites (Sun et al., 2020; Wei et al., 2022). Understanding the molecular mechanisms underlying cold acclimation, including the ones at the transcriptional level, can contribute to improving tolerance of high-yield king grass under chilling and freezing stress.

In this study, time-course RNA sequencing (RNA-seq) analysis was used to explore the dynamic changes in the transcriptome landscape of king grass exposed to cold temperatures (4°C) for varying durations. Key objectives were to 1) perform and analyze the transcriptome to obtain a global gene expression profile at nine time points, 2) identify differentially expressed genes (DEGs) during the cold stress, and 3) determine specific biological processes involved in cold stress, and 4) identify the transcriptional factors (TFs) associated with cold tolerance in king grass. Our results revealed the transcriptional patterns during short-term cold treatment and provided novel insights into how the forage was affected by cold stress.

## Materials and methods

### Plant growth and cold treatment

Tropical region-originated king grass (*Pennisetum purpureum* Schumacher × *P. americanum*) variety Reyan NO.4 with more than five years of cold acclimatization and located in the temperate zone of Sichuan province, China, was selected for this study. Following vegetatively propagation, the stalks were grown in a glasshouse (photoperiod of 16 h light/8 h, dark cycle) at ~20°C for 12 weeks. To standardize plant height and remove older tissue, the stalks were cut (12 cm above the soil level) twice in a glasshouse and another time prior to its transfer to a controlled environment chamber. The seedlings at a uniform growth stage post-cutting were cultivated in a plant growth chamber (at 25°C under 16 h light/8 h dark cycle) for three weeks and were used for cold treatment at 4°C at the three-leaf stage. For each cold treatment, three individual plants were sampled, and the entire leaf blade, from the tip to the base, were then collected from control (CK) and cold-treated plants at nine time points (0 h, 0.5 h, 1 h, 3 h, 6 h, 12 h, 24 h, 48 h and 72 h) post-cold exposure. Each cold treatment was repeated three times, providing three biological replicates for every time point. The collected samples were immediately frozen in liquid nitrogen for RNA preparation.

### RNA extraction, library preparation and sequencing

Each sample was ground separately in liquid nitrogen. Total RNA was extracted using the RNA prep Pure Plant Plus Kit (Polysaccharides & Polyphenolics-rich) (Tiangen, Beijing, China). Then, agarose gel electrophoresis and Nanodrop 2500 (Thermo Fisher Scientific, US) were used to determine the quality and quantity of each RNA extract. High quality RNA extract was used for single-molecule real-time (SMRT) and next-generation RNA sequencing.

For the PacBio SMRT sequencing, total RNA from different time points was pooled in equal amounts, and 2 μg of the pooled RNA sample was used for cDNA synthesis and SMRT library construction. First-strand cDNA was synthesized using the SMARTer PCR cDNA Synthesis Kit and amplified by polymerase chain reaction (PCR). Next, 1–6 kb fractions were collected. To obtain a sequencing library, PCR amplification of full-length cDNA, end-repair of full-length cDNA, connection of the SMRT dumbbell linker, and exonuclease digestion were performed. After the library was qualified, full-length transcriptome sequencing was performed using the PacBio platform (BioMarker, China). The PacBio raw bam file was deposited in the National Center for Biotechnology Information (NCBI) Sequence Read Archive (SRA) database (SRA; BioProject Accession: PRJNA1126330, BioSample Accession: SAMN41985243).

For Illumina sequencing, mRNA from 27 samples was added to the fragmentation buffer and cut into short fragments. Using mRNA as a template, cDNA was reverse-transcribed using six-base random primers. The double-stranded cDNA samples were purified, end-repaired, added with poly(A) tails, and then ligated to sequencing adapters to create cDNA libraries. Sequencing was conducted using the Illumina platform (BioMarker, China). The raw reads were deposited in the NCBI SRA database (BioProject Accession PRJNA1126330, BioSample Accession: SAMN41938665~SAMN41938691).

### Statistics, quality control, and annotation of raw sequencing data

The raw subreads from the PacBio platform were analyzed using the Iso-Seq3 pipeline (https://github.com/PacificBiosciences/IsoSeq). The pipeline included several steps: generation of circular consensus sequence (CCS) reads, classification of full-length (FL) reads, and clustering of FL non-chimeric (FLnc) reads. CCS reads were generated from the subreads BAM files using CCS (v6.2.0) with a minimum quality threshold of 0.9 (–min-rq 0.9) and a minimum of three passes (full passes ≥3). FL transcripts were identified as sequences containing both 5′ and 3′ cDNA primers and a poly(A) tail. Lima (v2.1.0) was used to remove the primers, and IsoSeq3 Refine was employed to trim poly(A) tails. High-quality FL consensus sequences were obtained using the ICE (Iterative Clustering and Error Correction) algorithm, with a post-correction accuracy threshold above 99%. Iso-Seq high quality FL transcripts for removing redundancy using cd-hit (v4.8.1, identity > 0.99). The Proovread software (v2.14.1) was subsequently applied to correct low-quality consistent sequences using Illumina RNA-seq data. Finally, the integrity of the transcripts was evaluated using Benchmarking Universal Single-Copy Orthologs (BUSCO, v5.8.2) to estimate the completeness of conserved genes across related species. This process ensured high-quality data for downstream analyses.

The transcripts were subjected to BLASTx queries (v2.16.0, https://ftp.ncbi.nlm.nih.gov/blast/executables/release/) with an e-value < 0.00001, based on the priority order of NCBI non-redundant (NR) protein sequences, SwissProt (http://ftp.ebi.ac.uk/pub/databases/swissprot), Gene Ontology (GO, http://geneontology.org), Kyoto Encyclopedia of Genes and Genomes (KEGG) (Kanehisa and Goto, 2000), and EuKaryotic Orthologous Groups (KOG) databases. To identify the gene family, the Hmmer v3.3.1 software (Finn et al., 2011) and Pfam (http://pfam.xfam.org) were used. Only one coding sequence (CDS) per transcript was produced as an output by setting the “–single_best_only” parameter and running a homology search against the UniProt database.

### Structure analysis of reference transcriptome

Simple sequence repeats (SSRs) in the transcriptome were identified using MISA v2.1 (http://pgrc.ipk-gatersleben.de/misa/) to detect and annotate SSR loci based on default criteria, ensuring reliable detection for downstream analyses. Candidate coding sequences (CDSs) were identified using TransDecoder v5.5.0 (https://github.com/TransDecoder/TransDecoder/releases) by applying the following criteria: a minimum length open reading frame (ORF) was detected; the log-likelihood score, computed by GeneID software, was greater than zero; the highest coding score was in the first reading frame; if one ORF was encapsulated by another, the longer ORF was reported, though multiple ORFs were allowed for transcripts to account for operons or chimeras; and optionally, the putative peptide matched a Pfam domain above the noise cutoff score. Alternative splicing (AS) events were identified using Iso-Seq™ data by performing an all-vs-all BLAST search with high identity thresholds. Candidate AS events were defined by BLAST alignments meeting the following criteria: the alignment contained two high-scoring segment pairs (HSPs) in the same orientation, with one sequence continuous or overlapping by less than 5 bp and the other containing a distinct “AS Gap” that exceeded 100 bp and was located at least 100 bp from the transcript ends. Long non-coding RNAs (lncRNAs) were identified using a combination of CPC2 v2.0, CNCI v2.0, CPAT v3.0.4, and Pfam v35.0 to differentiate non-protein-coding RNA candidates from protein-coding RNAs. Transcripts longer than 200 nt with more than two exons were selected as initial lncRNA candidates and further screened using these tools to filter out transcripts with coding potential or conserved protein domains.

### Identification of DEGs

To analyze differentially expressed genes (DEGs), we primarily relied on Illumina RNA-seq data, which were generated by selectively capturing polyA-tailed transcripts. This ensured that the sequenced transcripts were predominantly protein-coding. The expression levels of these Illumina-generated transcripts were quantified by mapping them to the Iso-Seq-based reference transcriptome, which was used as a comprehensive annotation resource. By combining the precision of polyA-enriched Illumina data with the detailed annotation from Iso-Seq, we ensured that our DEG analysis focused on biologically relevant coding transcripts while benefiting from the full-length transcript information provided by Iso-Seq.

The raw reads from Illumina sequencing were processed by removing adapter and primer sequences using TrimGalore (v0.6.10), as well as filtering out low-quality reads, to generate high-quality clean reads using FastQC (v0.12.1) and Trimmomatic (v0.39) for downstream analyses. Clean reads were mapped to the reference sequence using the STAR software (v2.7.11b) to obtain the location information of the transcripts (Dobin et al., 2013). The fragments per kilobase of transcript per million fragments mapped (FPKM) were calculated using the RSEM software (v1.3.1) and used to compare the expression levels of the transcripts (Li and Dewey, 2011). A principal component analysis (PCA) was performed using prcomp utilities in the R package. Differential expression analysis was performed using DEGseq2 (v1.46.0) in the R package with the FPKM values (Love et al., 2014). Genes were considered to be differentially expressed if they met the following criteria: |log2^(fold change)^ | > 1 and false discovery rate (FDR) < 0.05. KEGG enrichment analysis of DEGs was performed using the TBtools software (v2.148, https://github.com/CJ-Chen/TBtools) based on KEGG annotations of full-length transcripts and plant KEGG background (Kanehisa and Goto, 2000). The name of the enrichment pathway, p-value, and gene number were visualized using the ggplot2 package (v3.4.0) (Wilkinson, 2011).

### Co‐expression network analyses

Weighted Gene Co-Expression Network Analysis (WGCNA, 1.71) package was used in R for co-expression network analyses (Langfelder and Horvath, 2008). A convenient one-step network construction and module detection function were used to generate the co-expression gene network and identify each module with distinct functions. The optimal soft threshold was set at 22, where the fitting curve approached 0.9. Similar modules with a height cutoff value of 0.25 were combined, and the threshold of the number of genes in each module was set to 100. To identify the modules that significantly correlated with cold treatment time, we performed Pearson’s correlation analysis and computed the Student’s asymptotic *P*-value of each module at all cold treatment time points. All co-expressed DEGs were grouped into time point-specific modules. Each network was visualized using Cytoscape (v.3.7.2) (Shannon et al., 2003).

### Identification of protein kinases (PKs), transcription factors (TFs), and transcriptional regulators (TRs)

To predict protein kinases (PKs), transcription factors (TFs), and transcriptional regulators (TRs) in king grass, we utilized iTAK (v1.7), a widely used tool for plant transcriptional regulatory protein identification. iTAK integrates data from the Plant Transcription Factor Database (PlnTFDB) and Kinase Classification Database (KinaseDB) to classify and annotate PKs, TFs, and TRs. The transcript sequences from our reference transcriptome were analyzed using iTAK with default parameters. TFs were identified based on the presence of conserved DNA-binding domains, while TRs were annotated using their known functional domains and interaction motifs as defined in PlnTFDB. Similarly, PKs were predicted by identifying conserved protein kinase domains and classified into families according to KinaseDB rules. Each family of PKs, TFs, and TRs was further categorized and analyzed based on sequence similarity and domain characteristics. The iTAK-based prediction provided a comprehensive overview of regulatory proteins in the transcriptome, allowing us to analyze their differential expression under cold stress.

### Quantitative real-time-polymerase chain reaction (qRT‐PCR) assay

For the qRT-PCR assay, total RNA was extracted from the samples using a method similar to that adopted for RNA-seq. The extracted RNA was treated with DNase to remove genomic DNA contamination and subjected to reverse transcription for the synthesis of the first cDNA strand. qRT-PCR was conducted using the SYBR mix. Actin was used as an internal control. The gene primers were designed and shown in **Supplementary Table S1**. The relative expression levels of genes were calculated from three independent biological replicates using the 2^−ΔΔCT^ method (Livak and Schmittgen, 2001).

## Results

### Construction of full-length reference transcriptome of king grass

King grass has no available reference genome sequences yet. To establish its transcriptional profiles in response to cold stress, we constructed one SMRT library comprising one whole tissue culture plantlets (F01) and a mixed sample of 26 king grass seedlings subjected to low temperature for varying durations (F02). The libraries were sequenced each with three cells, yielding 60.36 and 42.02 Gb clean data for F01 and F02, respectively. A total of 671,808 and 361,660 CCSs (including 565,567 and 285,119 full-length reads non-chimeric (FLNC) were identified, and 163,949 and 94,995 high-quality non-redundant FLNC were identified after polishing using RNA-seq reads, clustering and demultiplexing of full-length transcripts in F01 and F02, respectively (**Table 1**). After merging FLNC transcript lists from the two libraries and conducting a redundancy analysis, we identified 57,068 unique transcripts. We simultaneously conducted Illumina RNA-seq of 26 samples for error correction and mapped all reads to full-length transcripts with a mean mapping rate of 84.08%, with 43.12% uniquely mapped reads and 37.51% of reads mapped to multiple loci. We identified 1,124 AS events and predicted 17,418 SSR, 7,321 lncRNA, and 23,573 complete CDSs in the full-length transcriptome (**Supplementary Figure S1**). Functional annotation of the transcripts was conducted using nine different public databases (COG, gene ontology (GO), KEGG, KOG, Pfam, Swissprot, TrEMBL, eggNOG and NR; **Supplementary Figure S2**). The results showed that about 83.56% of the transcripts (n = 47,685) had homologs with significant hits (E-value cutoff: 1e-05) in the databases, which was higher than previous reports (Zhao et al., 2019) of RNA-seq assembly sequences in king grass (40.15%, n = 146,650).

**Table 1:** PacBio sequencing data statistics.

	F01	F02
cDNA size	1-6 kb	1-6 kb
CCS number	671,808	361,660
Read Bases of CCS	942,127,440	570,529,164
Mean Read Length of CCS	1,402	1,577
Mean Number of Passes	40	42
Number of filtered short reads	0	0
Number of Full-length non-chimeric (FLNC)	565,567	285,119
FLNC percentage	84.19%	78.84%
Number of consensus isoforms	163,994	95,018
Average consensus isoforms length	1,531	1,463
Number of high-quality isoforms	163,949	94,995
Percent of high-quality isoforms (%)	99.97%	99.98%

### Temporal gene expression during different cold time points

**Figure 1:**
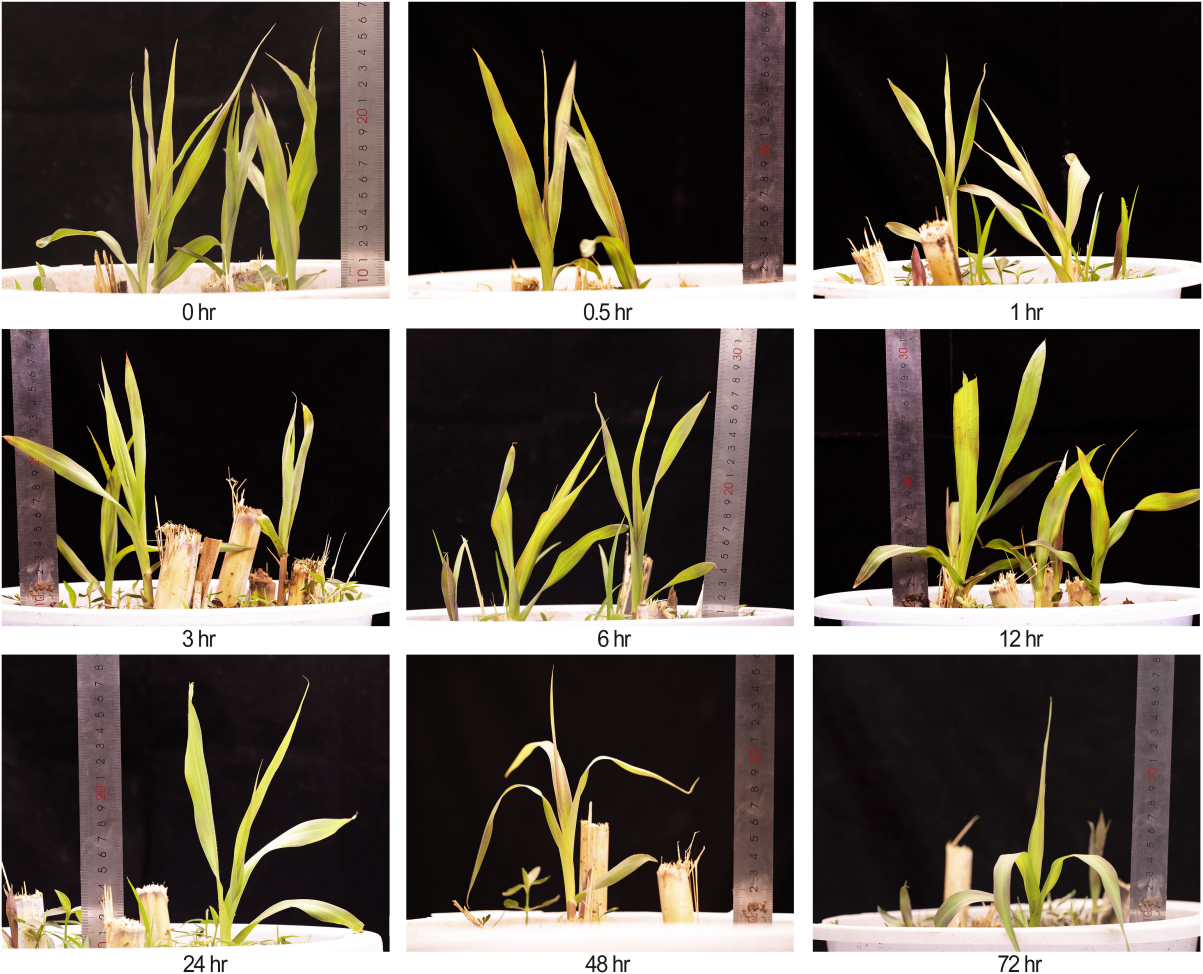
Phenotypes of king grass at nine time points (0.5 to 72 h) under cold stress at 4°C, including the untreated control (0 h). A ruler was placed to indicate plant height. Due to destructive sampling, the number of plants decreased at later time points.

**Figure 2:**
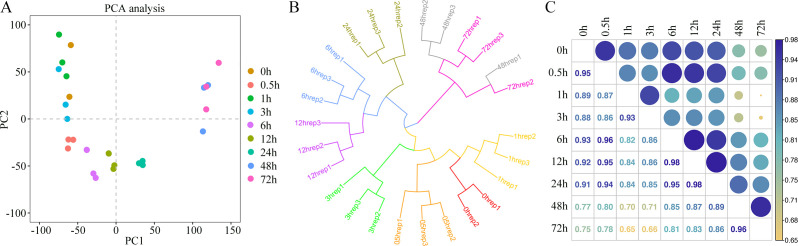
Transcriptome analysis of king grass at different times post-cold treatment. **(A)** Principal component analysis (PCA) of gene expression profiles before and after cold treatment. Each point represents one treatment for gene expression profiling. **(B)** Hierarchical clustering analysis (HCA) of gene expression values of all samples. **(C)** Pearson’s correlation coefficient of gene expression values of all samples.

To investigate the transcriptional changes in king grass seedlings under cold stress, we conducted RNA-seq over nine time points post-cold treatment (**Figure 1**). One of the CK sample replicates was removed due to technical errors during sequencing. In the remaining samples, the Pearson correlation coefficients between the biological replicates were statistically significant (correlation coefficients > 0.9, *P* < 0.001), indicating that the transcriptome data were suitable for the analysis of time point-specific gene expression. The clean reads of 26 samples were mapped to the full-length reference transcriptome, resulting in the identification of 57,068 genes. The number of expressed genes and their FPKM in each sample were also analyzed. We used PCA to examine time point-related transcriptional changes after cold treatment and observed that the profiles at different time points varied significantly, separating along the first coordinate (**Figure 2A**). Since we observed an intersection of some dots between close time points in PCA, we conducted hierarchical cluster analysis (HCA), which revealed that the expression patterns of genes formed three distinct clusters with the time points. The CK and groups with short-term cold treatments (0, 0.5, 1, and 3 h) were closest to clustering, although the 3-h sample was slightly clustered away from the 1-h sample. This result suggested no significant change, at least within an hour of cold treatment, in the expression patterns of the king grass genes. Furthermore, the 6-h, 12-h, and 24-h samples clustered together but were separated at their respective time points. However, the samples subjected to long-term cold treatments (48 and 72 h) were the closest to the clustering. These two samples exhibited similar intrinsic gene expression profiles, which were farthest from the samples subjected to short-term cold treatments (**Figure 2B**). The Pearson correlation coefficients indicated that the 48-h and 72-h samples were distant from the samples subjected to clod treatment for less than 3 h (correlation coefficients < 0.8), and the 6 h~12 h samples (correlation coefficients < 0.9), which was consistent with the PCA and HCA results (**Figure 2C**). These results suggested the expression of the king grass genes was affected by the time spans under cold stress and could be classified as early (before 3 h), middle (6~24 h) and late responses (48 and 72 h).

**Figure 3:**
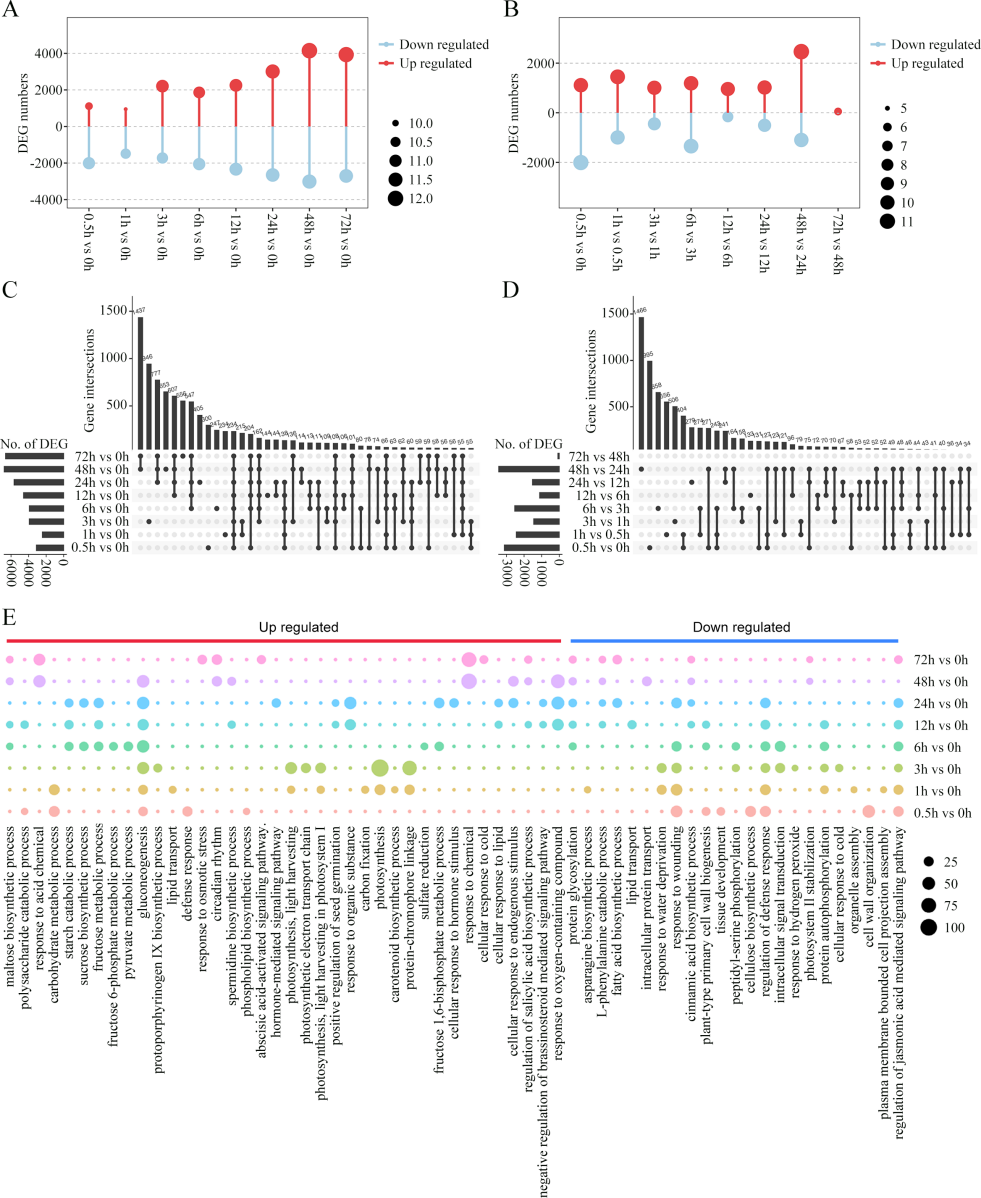
Differentially expressed genes (DEGs) during cold treatment in king grass. **(A, B)** Bar graph showing the total number of differentially upregulated (red) and downregulated (blue) genes at each pairwise comparison between the cold-treated samples and CK **(A)** and between the latter time-point samples and the previous time-point samples **(B)**. **(C, D)** The UpSet R plot showing numbers and interactions of DEGs between the cold-treated and CK samples **(C)** and between the latter time-point samples and the previous time-point samples **(D)**. The horizontal bars show the number of DEGs in each set, while the vertical bars represent the intersection size. Dark spheres indicate which sets are represented in the vertical bar. The total numbers of upregulated and downregulated genes at each of the time points are shown on the left. **(E)** Temporal ordering of biological processes during cold treatment as identified using gene ontology (GO) enrichment analysis.

HCA provided a global view of the temporal alterations in the gene expression profiles during the cold treatment. A comparison of the different treated samples revealed 13,056 DEGs. We compared the gene expression patterns and identified the up- and down-regulated DEGs between the cold-treated and control samples (**Figure 3A**). In general, 17.95%~52.80% of the DEGs were identified in different treated samples. The number of DEGs tended to increase along with the cold stress duration, with most DEGs in the 48-h sample compared with the 0-h sample. Thus, the number of DEGs was larger at the late response time points compared to the early response time points. Furthermore, we compared the number of DEGs in the latter time-point samples with those in the earlier time-point samples. As shown in **Figure 3B**, the sample corresponding to the first time point of each response stages, that is, 0.5, 6 and 48 h, showed substantially more DEGs in comparison to the sample in the previous stage, suggesting a change in gene expression patterns during early, middle, and late responses. Next, the DEG sets between each time point were compared and visualized. UpSet visualization was used to observe the overlapping DEGs between two or multiple time points. The plot showed that many DEGs were comparison set-specific, including 946, 653, and 556 genes in sets 3 h vs. 0 h, 48 h vs. 0 h, and 72 h vs. 0 h, respectively (**Figure 3C**). Notably, a large number of genes were combined as DEGs co-regulated in the middle and later response stages, with 48-h and 72-h samples sharing 1,437 DEGs within the later response stages, and 547, 607, and 777 DEGs common after 6, 12, and 24 h, respectively, across middle and later response stages. As for the DEGs shared among combinations of the latter time-point samples and the previous time-point samples, the majority of them were comparison set-specific (**Figure 3D**). Most unique DEGs (n = 1,466) were detected during the comparison between 48-h and 24-h samples, followed by 995 and 658 between 0.5-h and 0-h samples and 6-h and 3-h samples, respectively.

### Dynamic changes in king grass transcriptome in response to cold stress

To further analyze the dynamic changes in the gene expression profiles at different time points, we conducted GO enrichment analysis of the DEGs and observed a time-dependent shift in the GO terms, reflecting the different potential biological functions during the various stages of cold response (**Figure 3E**). Early responsive genes to cold stress that had high expression levels between 0.5 and 3 h post-cold treatments were mainly enriched in glycolipid metabolism and photosynthesis, including polysaccharide catabolic process, carbohydrate metabolic process, gluconeogenesis, lipid transport, phospholipid biosynthesis, light harvesting in photosystem I, photosynthetic electron transport chain, carbon fixation, and protein-chromophore linkage. The responsive genes in the middle response stage (6~24 h post-cold treatment) were mainly enriched in carbohydrate metabolism, such as maltose and sucrose biosynthesis, fructose metabolic process, and starch catabolic process. The enriched terms were also related to the positive regulation of seed germination and plant hormone pathways, including the negative regulation of the brassinosteroid (BR)-mediated signaling pathway. The genes exhibiting a constant upregulation during the relative long-term cold treatments (48 and 72 h) were related to response to cold stress, osmotic stress, and acid chemical and endogenous stimulus. Importantly, GO terms enriched by these upregulated genes were also relevant to hormone-mediated signaling pathways, such as the abscisic acid-activated signaling pathway, and the regulation of salicylic acid biosynthesis. However, the genes enriched in the regulation of the jasmonic acid-mediated signaling pathway were downregulated in later response stages.

**Figure 4:**
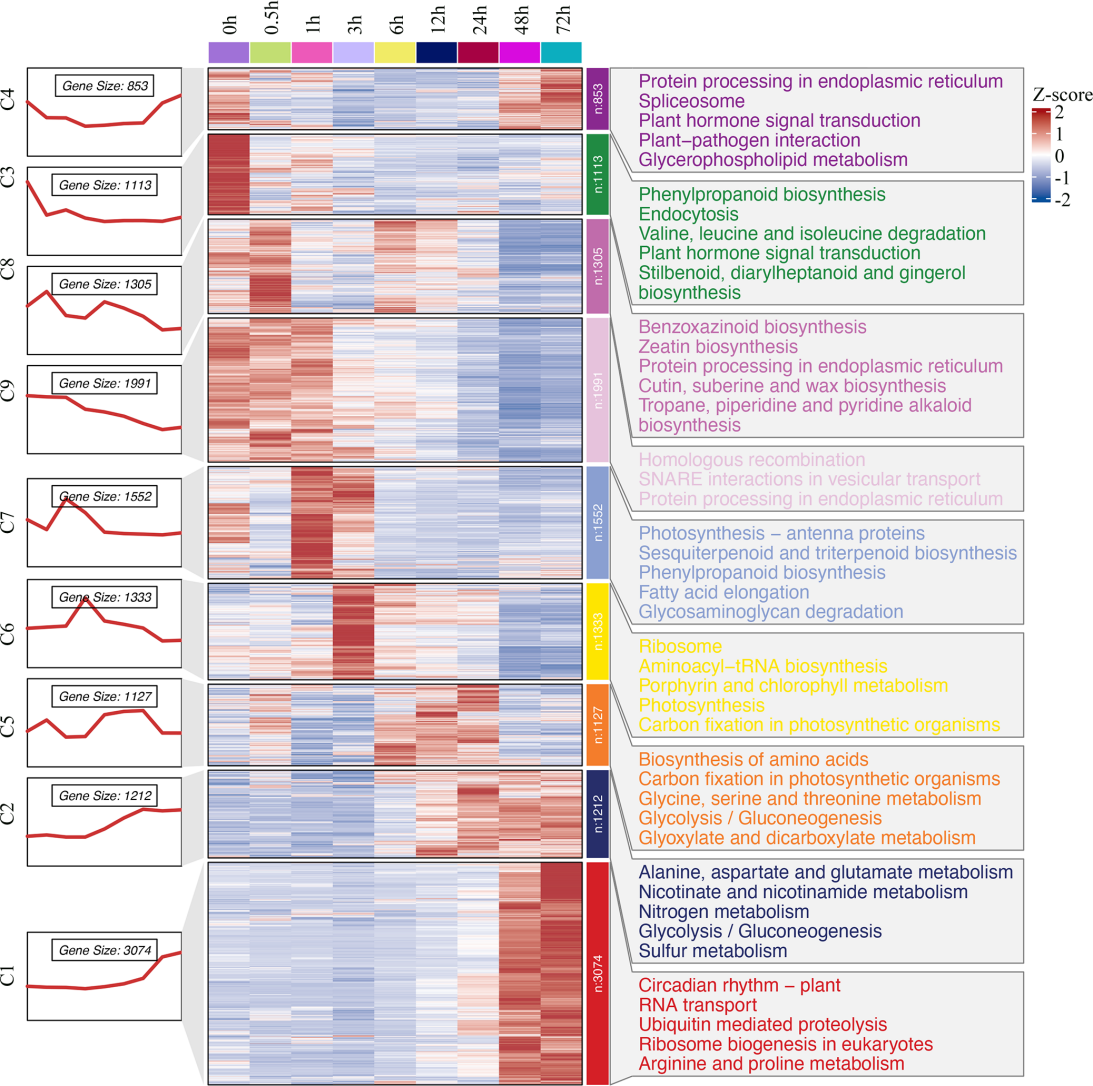
Hierarchical clustering and heat map of king grass DEGs and key GO terms. The numbers and expression patterns of genes in the different clusters are shown on the left part. Middle part: Heatmap showing the expression profiles of DEGs at each time point. The heatmap was generated through the k‐means clustering algorithm with a row‐wise Z‐score result of gene expression in the Cluster 3.0 software. The color key representing the standardized gene expression levels from high (red) to low (blue). Right part: GO-enriched biological processes for nine clusters.

Hierarchical clustering of the expression profiles showed progressive expression dynamics as a cold accumulation process. DEGs from the comparison sets were clustered into groups based on expression changes across time points. The results were visualized by a heatmap (**Figure 4**), which indicated a varying temporal expression pattern for king grass genes under cold stress. A total of nine co-expressed clusters were generated through the k‐means clustering algorithm, each of which contained a unique gene set comprising 853 to 3,074 members. These distinct patterns suggested a precise temporal transcriptional regulation corresponding to the different durations of cold exposure. Particularly, cluster 3 (n = 1,113), which mostly represented downregulated genes at all cold treatment stages, was enriched for GO terms related to the regulation of the jasmonic acid-mediated signaling pathway, regulation of defense response, etc. The clusters with upregulated genes in response to short-term cold stress, such as clusters 7 (n = 1,552) and 6 (n = 1,333), were enriched for GO terms associated with chlorophyll-binding, photosystem, chloroplast organization, and ribosome biogenesis, indicating photosynthesis was largely reduced by cold treatment. Along with prolonging cold treatment, the genes that upregulated specifically in middle response stages (6, 12, and 24 h) were clustered in cluster 5 (n = 1,127) and enriched for GO terms associated with photosystem repair and photoinhibition. In contrast, the clusters with genes upregulated in response to long-term cold treatments (sustainably upregulated after 12 h), such as cluster 2 (n = 1,212), were enriched for GO terms associated with nitrate assimilation, molybdenum ion binding, and nitric oxide (NO) biosynthesis. In addition, 3,074 genes were specifically upregulated in 48-h and 72-h samples, clustered in cluster 1, and enriched for GO terms associated with DNA-binding transcription factor activity, cellular response to cold, and response to acid chemical. These genes were critical for the response of king grass to late-stage cold stress. These results showed that the expression patterns of DEGs varied and corresponded to distinct biological functions depending on the duration of cold stress.

### Identification of differentially expressed TFs and protein kinases (PKs) in cold stress

**Figure 5:**
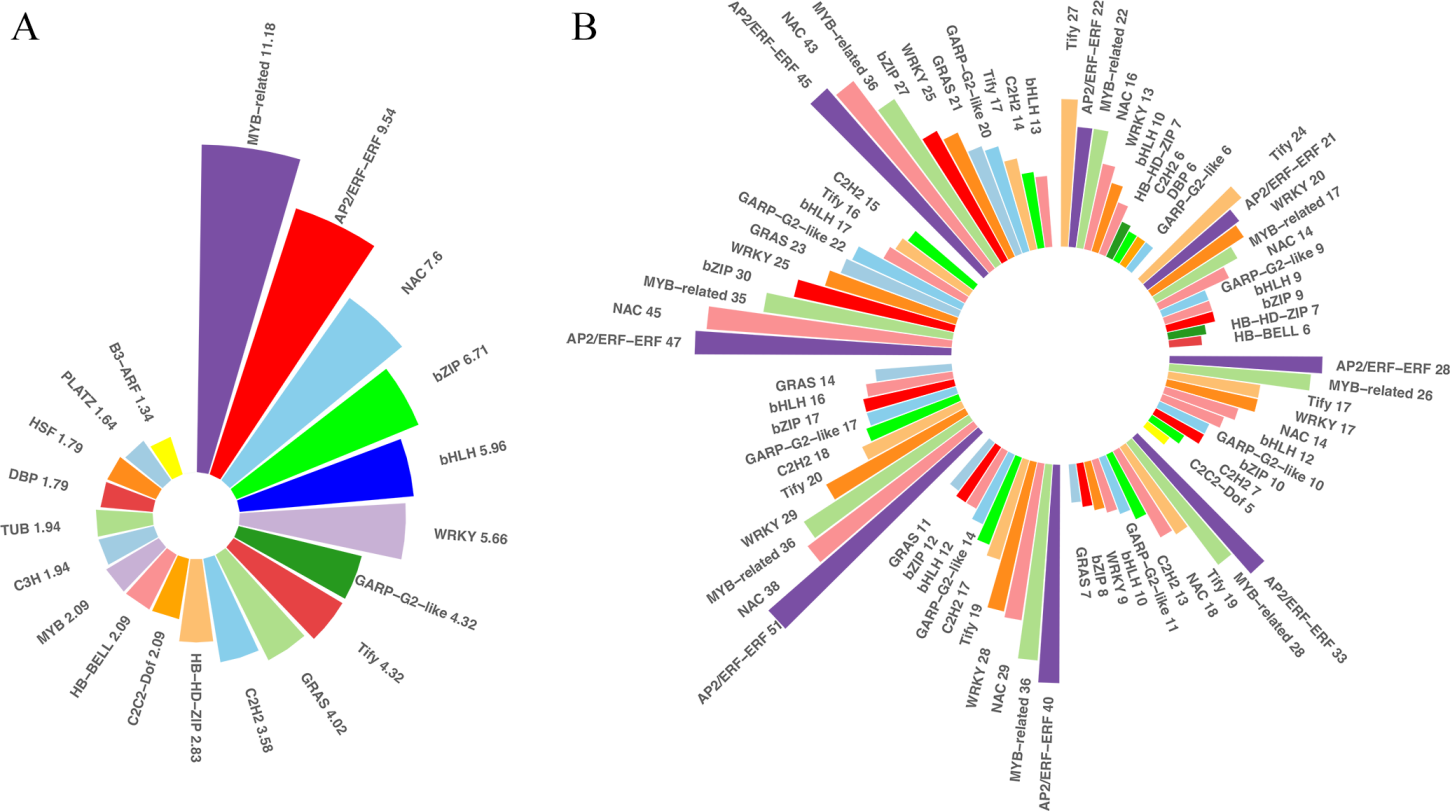
Differentially expressed transcription factors (TFs). **(A)** Bar plots showing the distribution of the top 20 differentially expressed TFs across the differentially expressed genes (DEGs). **(B)** Bar plots showing the distribution of the top five differentially expressed TFs at each time point.

TFs and PKs are important regulators of the responses to cold stresses. TFs and other transcription regulators (TRs) regulate spatial and temporal gene expressions. A plethora of transcriptional regulatory proteins have been identified and classified into families on the basis of sequence similarity. In total, 1,982 TFs were differentially expressed in our transcriptome sets under low temperatures. These TFs belonged to 52 families. The top 20 of the differentially expressed TF families were shown in **Figure 5**, and the families with the most abundant TFs were MYB-related (11.8%), followed by AP2-ERF (9.54%), NAC (7.6%), bZIP (6.71%), bHLH (5.96%), and WRKY (5.66%), etc. These TFs were distributed across the DEGs at different time points, indicating that TFs play important roles during prolonged cold treatment. In the very early response stage, Tify was the most abundant TF family among the differentially expressed TFs, but it descended to third and fourth most abundant in the middle response stages and ranked ninth at 48 and 72 h post-cold stress. In contrast, the number of AP2-ERF, MYB-related and NAC TFs were relatively abundant and stable at each time point, indicating that these families likely play important regulatory roles in both short- and long-tern cold stress. In addition to TFs recognizing cis-regulatory DNA sequences, additional proteins and other TRs are involved in protein-protein interactions underlying plant cold-stress responses. TRs of king grass were identified using iTAK, which is based on the rules of PlnTFDB. Aux/IAA, one of the key regulators of auxin responses in plants, was the most abundant TRs identified (12.57%), followed by GNAT (8.98%) and IWS1 (5.39%), etc. However, the TRs were not abundantly expressed at different stages of cold treatment, except for AUX/IAA family genes, which were relatively abundantly expressed in the middle to later response stages of cold treatment (**Supplementary Figure S3**). Similarly, we also observed variations in the proportions of down- and upregulated PKs under cold treatments. CHK1, belonging to the CAMKL family, was more upregulated than other PKs, accounting for 12.05% of all differentially expressed PKs and relatively abundantly expressed after 6 h of cold treatment. Another member of the CAMKL family, CDPK, was abundantly expressed in the early-response stage before 6 h of cold treatment (**Supplementary Figure S3**). Therefore, the calcium-dependent protein kinases were differentially expressed across all time points and exhibited important roles in response to cold treatment.

### Roles of hub TFs in cold stress

**Figure 6:**
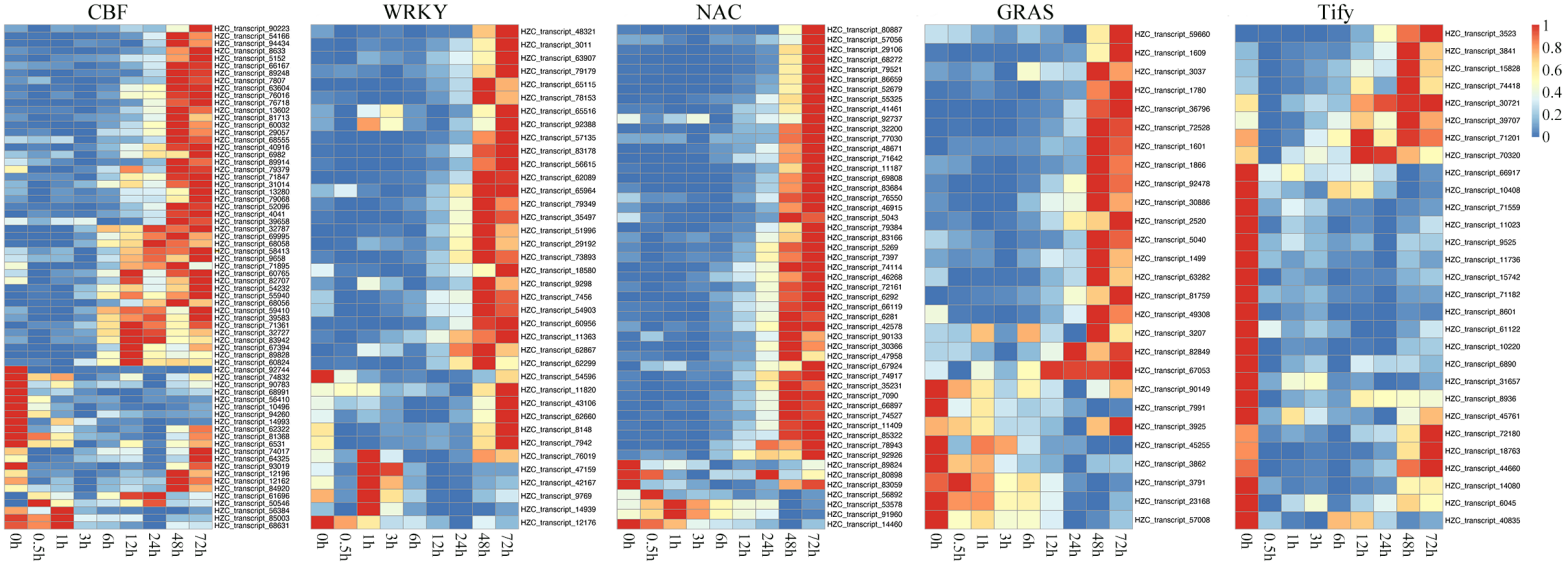
Heatmaps showing the expression patterns of key transcription factors (TFs) belonging to the CBF, WRKY, NAC, Tify, and GRAS families under cold treatment.

We identified some key TFs with regulatory relationships with each other that played important roles in regulating DEGs under cold treatment. In the respect, the classical CBF-dependent low-temperature signaling pathway in plants has been discussed relatively thoroughly. CBFs play a central role in cold acclimation and have been successively identified as important TFs located upstream of the cold-responsive genes (CORs, induced by low-temperature environment). Based on the rich knowledge base associated with ICE-CBF-COR in maize and the conservation of ICE-CBF-COR in Poaceae and protein sequence alignment, we identified the expression of 57 CBF1/2/3-related genes. As shown in **Figure 6**, the 46 orthologous genes encoding CBF1 and CBF3 were late in response to cold stress. Eventually, their transcript levels gradually increased after 12 h. The remaining 11 orthologous genes encoding CBF2 exhibited the opposite expression pattern, with decreased expression levels at the beginning of cold treatment. These findings were consistent with the results obtained in Arabidopsis, which showed that CBF2 negatively regulates CBF1 and CBF3. Importantly, the expression patterns of CBF1 and CBF3 were similar to those of other TFs, like WRKY, NAC, and GRAS, which were activated and upregulated in the later response stage (48 and 72 h). In contrast, Tify showed a negative regulatory relationship with the duration of cold treatment.

**Figure 7:**
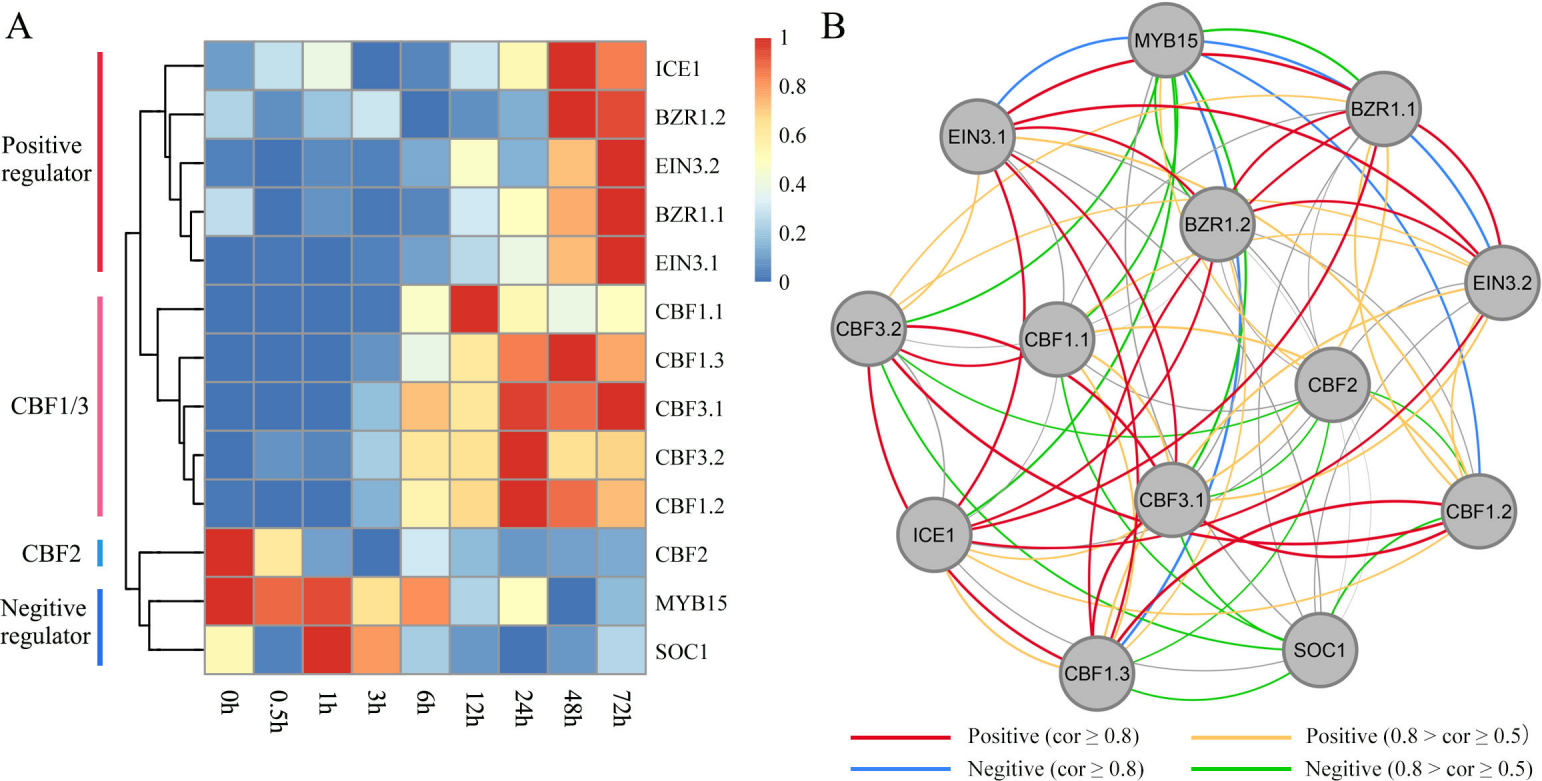
Identification of ICE-CBF-COR genes in king grass and visualization of their co-expression network. **(A)** Heatmaps showing the expression patterns of 13 ICE-CBF-COR genes. The red and blue colors indicate high and low expression levels, respectively. **(B)** Visualization of the co-expression networks of the 13 ICE-CBF-COR genes.

Based on WGCNA, we identified some important hub TFs that co-expressed with each other. We assessed whether they exhibited regulatory relationships with each other and played important roles in the regulation of the ICE-CBF-COR signaling module under cold treatment. Based on the rich research background associated with ICE-CBF-COR in Arabidopsis, we identified the CBF1/2/3-related genes, including ICE1, MYB15, SOC1, EIN3 and BZR1 (**Figure 7A**). We performed qRT-PCR with these thirteen target genes to confirm the RNA-seq data. The fold changes of gene expression in the target genes had a similar trend with those from RNA-seq analysis (R2 = 0.9232, **Supplementary Figure S4** and **Supplementary Table S2**), which indicated a high reliability of RNA-seq data. We then identified positive and negative regulators of the orthologous genes according to their expression patterns under cold treatment. In Arabidopsis, CBF2 negatively regulates CBF1and CBF3expression. ICE can directly bind to CBF promoters and contribute to the regulation of constitutive CBF expression, particularly of CBF1 and CBF3. MYB15, SOC, and EIN3 negatively regulate ICE1 expression. BZR1 positively regulates freezing tolerance via CBF-dependent and independent pathways. In our Poaceae king grass dataset, we also observed that CBF1 and CBF3 were clustered together while being distant from CBF2, indicating that the functions of CBF1/2/3 were conserved across monocotyledonous and dicotyledonous plants. Genes showing positive or negative regulatory relationships with CBFs also exhibited similar effects in king grass. For instance, MYB15 and SOC1 were downregulated when CBF1 and CBF3 were upregulated during cold treatment. BZR-mediated output from the brassinosteroids (BRs) signaling pathway contributes to cold tolerance through the upregulation of the CBF cold-response pathway. However, two EIN3 orthologs were upregulated along with prolonging cold treatment, which was inconsistent with the previous report in Arabidopsis (Shi et al., 2012). We further established a co-expression regulatory network and visualized the mutual regulatory relationship between these 13 genes (**Figure 7B**). The positive and negative regulatory relationships were also shown in the network, providing certain insights into the correlation between the related genes in king grass and the ICE-CBF-COR signaling module at low temperatures.

## Discussion

To cope with various environmental stresses adversely affecting their normal growth and development, plants employ several changes in their morphology, physiology, biochemistry, metabolism, genetics, and epigenetics (Georgieva and Vassileva, 2023). Global transcriptional changes in response to cold stress and molecular mechanism underlying cold-induced reprogramming of gene expression have been intensively investigated in various plant species. However, there is a lack of similar studies on king grass. Although the whole genome of king grass has not yet been assembled, full-length transcriptome sequencing of king grass has made it feasible to identify stress-resistance genes at the gene expression level. In this study, we determined the regulatory mechanisms underlying cold stress response in king grass by performing comprehensive multiple time point transcriptomic analyses and identified the up- and down-regulated genes at each time point, including cold-responsive genes, TFs, and PKs.

We identified several DEGs, suggesting that the expressions of genes in king grass changed dynamically during cold exposure. The number of comparisons set-specific DEGs fluctuates across different time points, suggesting that the plant’s response to cold stress is highly dynamic and occurs in distinct phases. At early time points (0.5 h and 1 h), fewer specific DEGs were detected (300 and 234, respectively), indicating the activation of a limited set of rapid-response genes to perceive and signal cold stress. A sharp increase in specific DEGs was observed at 3 h (946), likely reflecting a peak in transcriptional reprogramming where stress-responsive pathways are strongly induced. This was followed by a decline at 6 h (247) and 12 h (144), possibly as the plant shifts from acute responses to early adaptation mechanisms. Interestingly, the number of specific DEGs increased again at 24 h (405) and 48 h (653), suggesting the activation of genes involved in sustained responses, such as metabolic adjustments and structural modifications, to cope with prolonged cold stress. The subsequent slight decrease at 72 h (556) may indicate stabilization of the transcriptional response as the plant reaches a new homeostasis. These findings demonstrate that the transcriptional response to cold stress is not a simple linear process but rather involves multiple phases characterized by dynamic shifts in gene expression profiles.

We divided DEGs into nine clusters based on their expression pattern, and found that the genes in clusters 1 and 2, which were highly expressed at 48 and 72 h post-cold stress, were enriched and directly related to the cold stress response. We speculated that these DEGs might help to enhance the tolerance of king grass to late-stage cold stress. Especially the genes upregulated in response to long-term cold treatments (after 12 h) were enriched for GO terms associated with nitrate assimilation, nitrate reductase activity and NO biosynthesis, which are related to nitrogen availability. Many researchers have reported the relationship between nitrogen assimilation and cold tolerance in plants (Hassan et al., 2021; Ritonga and Chen, 2020; Soualiou et al., 2023). Generally, cold stress affects root system growth by reducing nitrogen uptake and translocation, limiting nitrogen availability in plants (Soualiou et al., 2023). Our results demonstrated that nitrogen assimilation, including NO production, was related to cold acclimation and response to long-term cold treatments. Previous reports have shown that nitrate reductase, which is usually associated with nitrogen assimilation, can mediate NO production from nitrite in an NAD(P)H-dependent manner and plays an important role in cold acclimation and freezing tolerance in Arabidopsis (Zhao et al., 2009). After cold stress, the ability of maize seedlings to recover increases via increased activities of nitrogen assimilation enzymes and improved redox homeostasis (Soualiou et al., 2023).

Notably, genes that were responsive to the early stages of cold stress in the current study were enriched in photosynthesis and phospholipid biosynthesis. These genes were upregulated in the first 3 h of cold treatment, which was inconsistent with the previous observations in Arabidopsis (Xi et al., 2020). In Arabidopsis, genes involved in photosynthesis and photosystem were downregulated during the cold treatments, indicating that photosynthesis was repressed in response to cold stress. However, we identified a brief upregulation of photosynthesis-related genes before the inhibition of the photosynthetic efficiency. We speculated that these early responsive genes are aimed at accumulating energy to cope with cold stress. It has been reported that plants with cold acclimation have a high photosynthetic capacity (Rapacz et al., 2008). As shown in **Figure 4**, the upregulation of early responsive genes involved in photosynthesis and phospholipid biosynthesis produced more energy to remodel chromatin structure to reprogram gene transcription, maintain membrane stability, facilitate endurance to cold injury, and accomplish all kinds of energy-dependent biological processes. For example, genes upregulated at 3 h or later were enriched for GO terms associated with ribosome biogenesis and rRNA processing, which is one of the most energy-demanding processes in the cell and is usually associated with cellular stress. In addition, we observed a significant downregulation of photosynthesis after 3 h post-cold treatment and the genes were enriched in photoinhibition within 6~24 h post-treatment, indicating that cold stress caused a downregulation of light absorption and a decrease in photosynthetic efficiency after energy accumulation, potentially protecting plants from photo-oxidative damage.

The expression of many TFs is also regulated in response to cold stress (Abdullah et al., 2022). In the present study, a total of 1,982 differentially expressed TFs, belonging to 52 TF families, were identified. The key TF families identified in the current study have also been identified as crucial in responses to cold temperatures in rice (Shen et al., 2014), *Zea mays* (Waititu et al., 2021), and *Triticum aestivum* L (Jiang et al., 2022), indicating that these TF families might have similar biological functions and regulatory roles in different plant species under cold stress. Moreover, we identified 13 genes in the ICE-CBF-COR signaling module and analyzed their expression at different time points under cold stress. We found both positive and negative regulatory factors and determined their regulatory relationships, which were generally consistent with the previous results on Arabidopsis (Barrero-Gil and Salinas, 2017; Kim et al., 2015). However, EIN3 was upregulated in the CBF cold-response pathway, which demonstrated that ICE-CBF-COR is not conserved between monocotyledonous and dicotyledonous plants. In addition, the orthologous genes of CBF1/2/3 in king grass were conserved to those in Arabidopsis both in terms of sequence similarity and regulatory function. However, previous studies have reported that CBF and COR genes are early-response genes playing an important role in short-term cold treatment in Arabidopsis (Gilmour et al., 1998). Our results demonstrated that CBFs and the related genes in the ICE-CBF-COR signaling module were middle- and late-response genes, with a significant upregulation after 24 h post-cold treatment. Their expression patterns might be associated with the high expressions of MYB15 and SOC1 in the early stages of cold stress. Cold stress upregulates MYB15, and the MYB15 protein interacts with ICE1 and binds to the MYB recognition sequence in the promoter sequence of CBFs, which reduces the expression of CBF genes during cold stress.

Based on our findings, we conclude that king grass employs a dynamic transcriptional reprogramming strategy involving energy accumulation, photosynthesis adjustment, and nitrogen assimilation to enhance cold tolerance. Key transcription factors and protein kinases play pivotal roles in mediating this response, while the ICE-CBF-COR signaling pathway exhibits unique temporal characteristics, underscoring differences in cold adaptation mechanisms between monocotyledonous and dicotyledonous plants.

We acknowledge that in this study, the untreated sample at 0 hours was used as the control for all cold stress treatment time points. While this approach is widely applied in transcriptomic studies on plant stress responses and has been utilized to identify key regulatory pathways and stress-responsive genes (Liu et al., 2022; Zhang et al., 2024), it may not fully account for developmental or physiological changes occurring independently of the cold stress over the time course. Ideally, time-matched controls (e.g., plants grown under normal conditions for 72 hours compared to 72-hour cold stress treatments) would provide a more precise reference, allowing for the separation of stress-specific transcriptional changes from those related to normal growth or development. We chose the 0-hour untreated sample as the baseline to align with the common practice in stress response studies and to simplify comparisons across multiple time points. However, we recognize that this methodology could introduce biases, particularly at later time points, where developmental divergence between treated and untreated samples might be more pronounced. Although applying complementary methods such as co-expression network analysis could further validate the findings and minimize potential limitations introduced by using a single baseline control, integrating time-matched controls alongside a 0-hour untreated baseline could enhance the resolution of stress-specific gene expression patterns in future studies.

## Data Availability

The data presented in the study are deposited in the National Center for Biotechnology Information (NCBI) Sequence Read Archive (SRA) database, BioProject accession number PRJNA1126330.

## Supplementary material

Supplementary Figure 1 Characteristics of the full-length reference transcriptome of king grass. **(A)** the types and the number of predicted SSR in the transcriptome. **(B)** prediction of lncRNA in four databases. **(C)** Distribution of predicted transcription factor (TFs) in the transcriptome. **(D)** Distribution of predicted lengths of protein sequences encoded by complete ORF regions.

Supplementary Figure 2 Transcript annotation into Nr database. **(A)** Nr identity distribution through sequence alignment. **(B)** The distribution of homologous species in Nr alignment results.

Supplementary Figure 3 Differentially expressed transcription regulators (TRs) and protein kinases (PKs). **(A, C)** Bar plots showing the distribution of the top 20 differentially expressed TRs and PKs across the differentially expressed genes (DEGs). **(B, D)** Bar plots showing the distribution of the top five differentially expressed TRs and PKs at each time point.

Supplementary Figure 4 Quantitative real-time PCR (qRT-PCR) validation the 13 ICE-CBF-COR genes of RNA-seq. FPKM values (left) and relative expression (right) of genes in different cold times.
