# Supplementary figures and images for: Dynamic changes in the transcriptome of tropical region-originated king grasses in response to cold stress
Source: Front Plant Sci. 2025 Feb 18;16:1511466. doi: 10.3389/fpls.2025.1511466 (PMC11876387; doi:10.3389/fpls.2025.1511466)

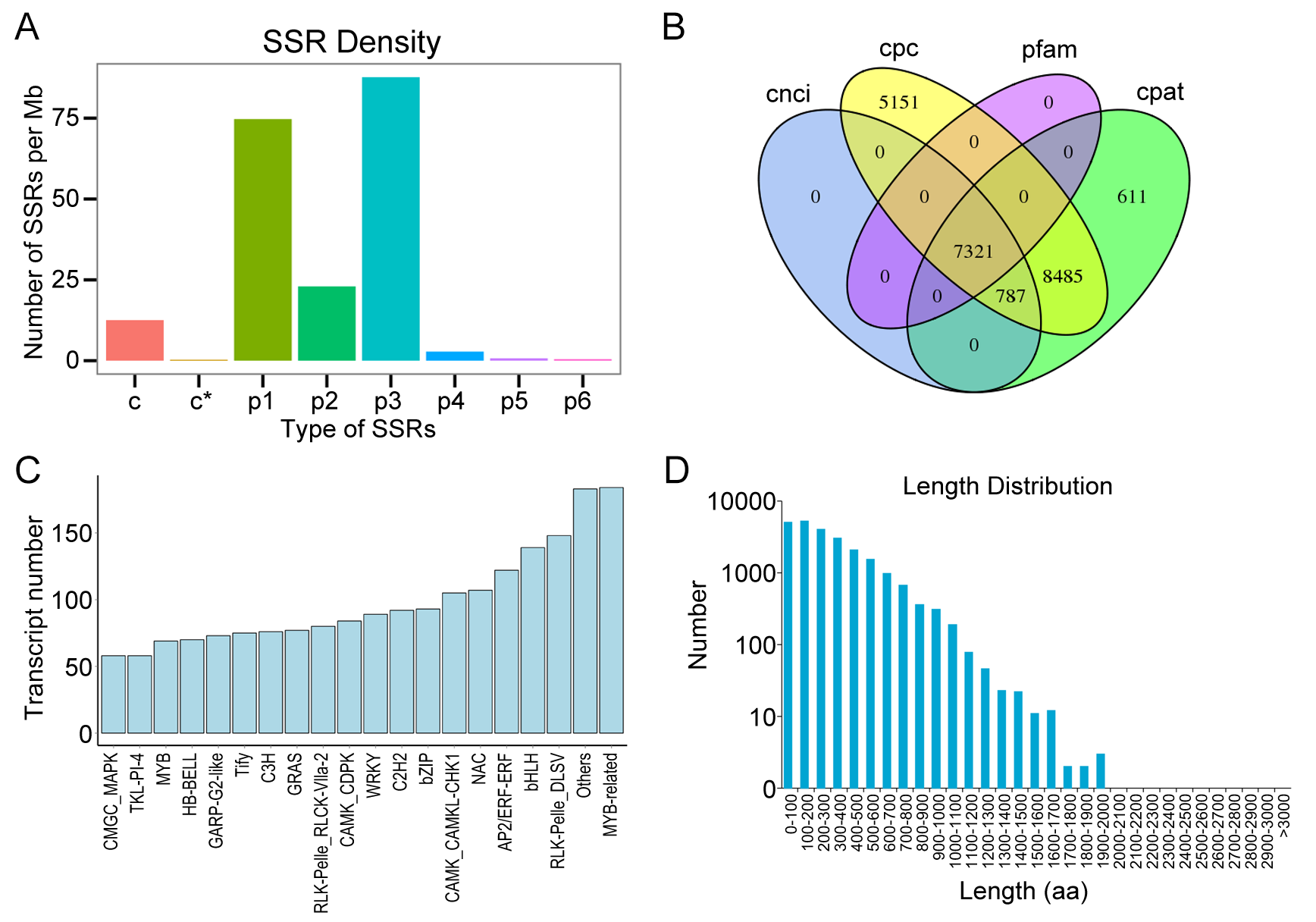

Supplement: Supplementary Figure 1 — Characteristics of the full-length reference transcriptome of king grass. (A) the types and the number of predicted SSR in the transcriptome. (B) prediction of lncRNA in four databases. (C) Distribution of predicted transcription factor (TFs) in the transcriptome. (D) Distribution of predicted lengths of protein sequences encoded by complete ORF regions. [file Image1.tif]

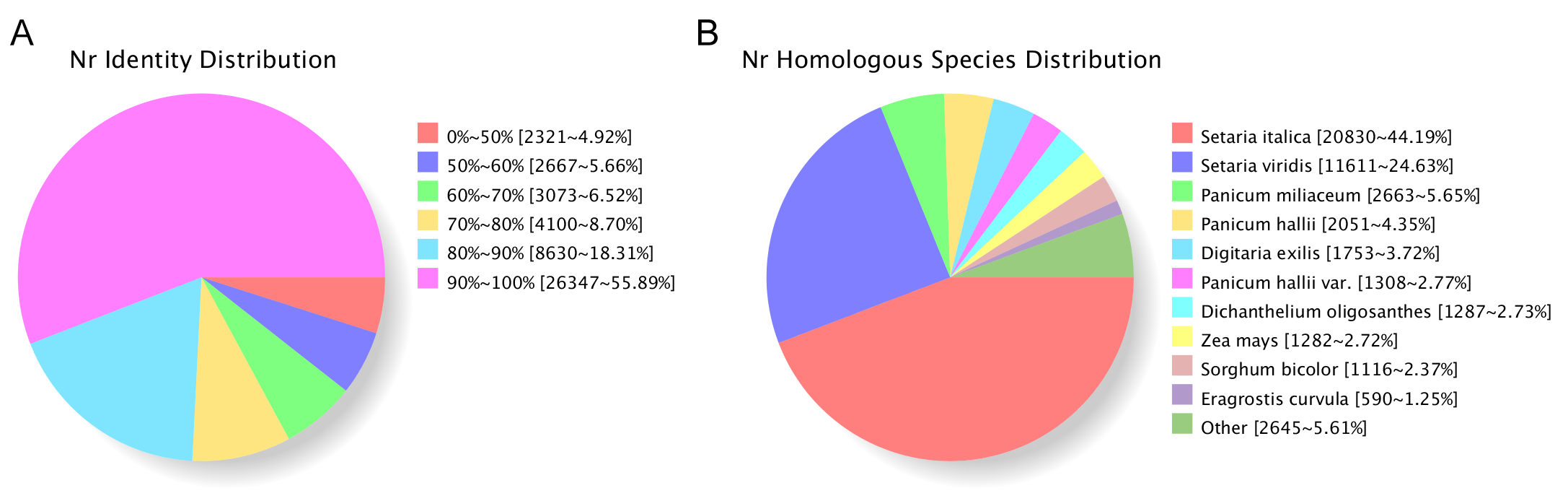

Supplement: Supplementary Figure 2 — Transcript annotation into Nr database. (A) Nr identity distribution through sequence alignment. (B) The distribution of homologous species in Nr alignment results. [file Image2.tif]

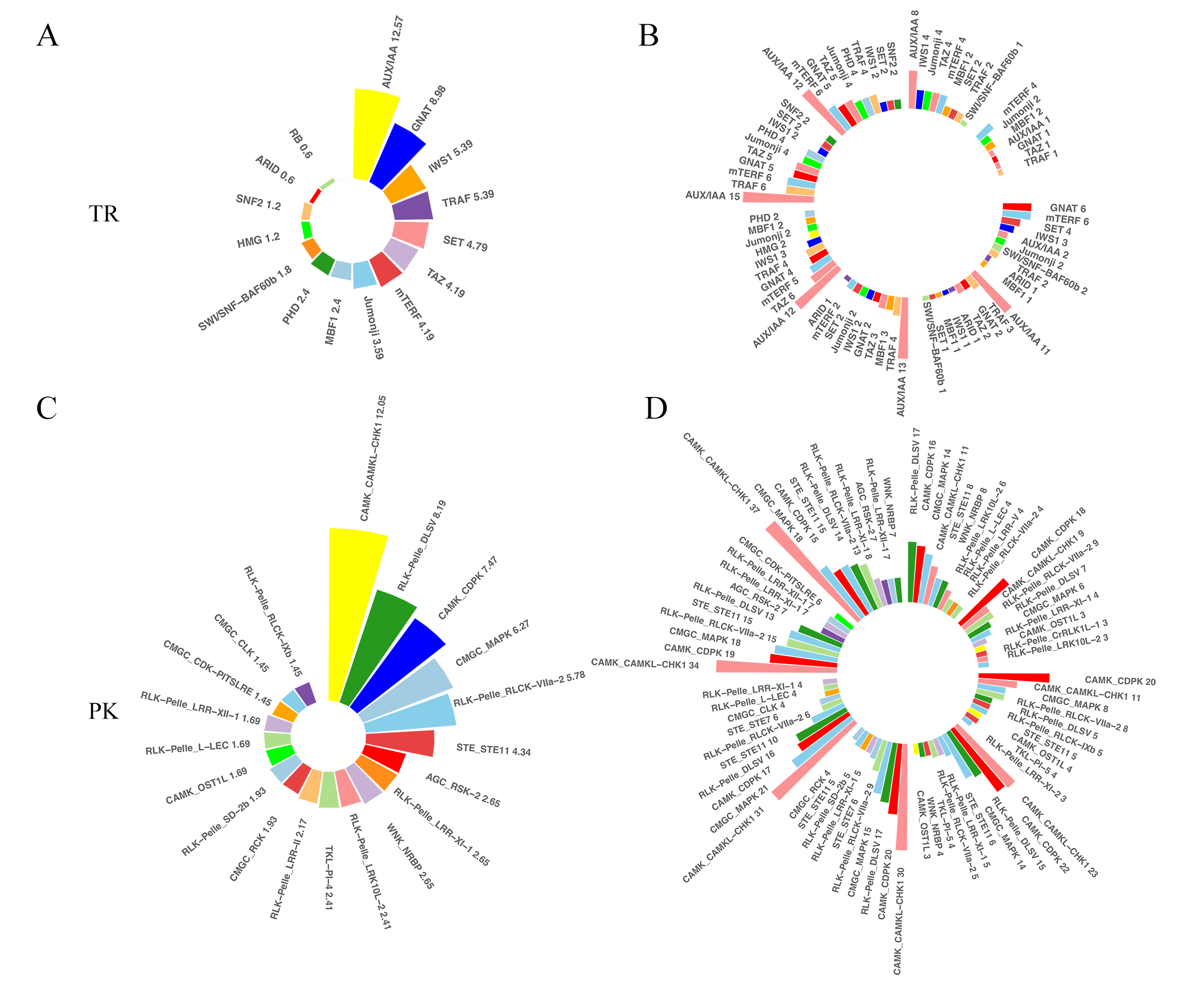

Supplement: Supplementary Figure 3 — Differentially expressed transcription regulators (TRs) and protein kinases (PKs). (A, C) Bar plots showing the distribution of the top 20 differentially expressed TRs and PKs across the differentially expressed genes (DEGs). (B, D) Bar plots showing the distribution of the top five differentially expressed TRs and PKs at each time point. [file Image3.tif]

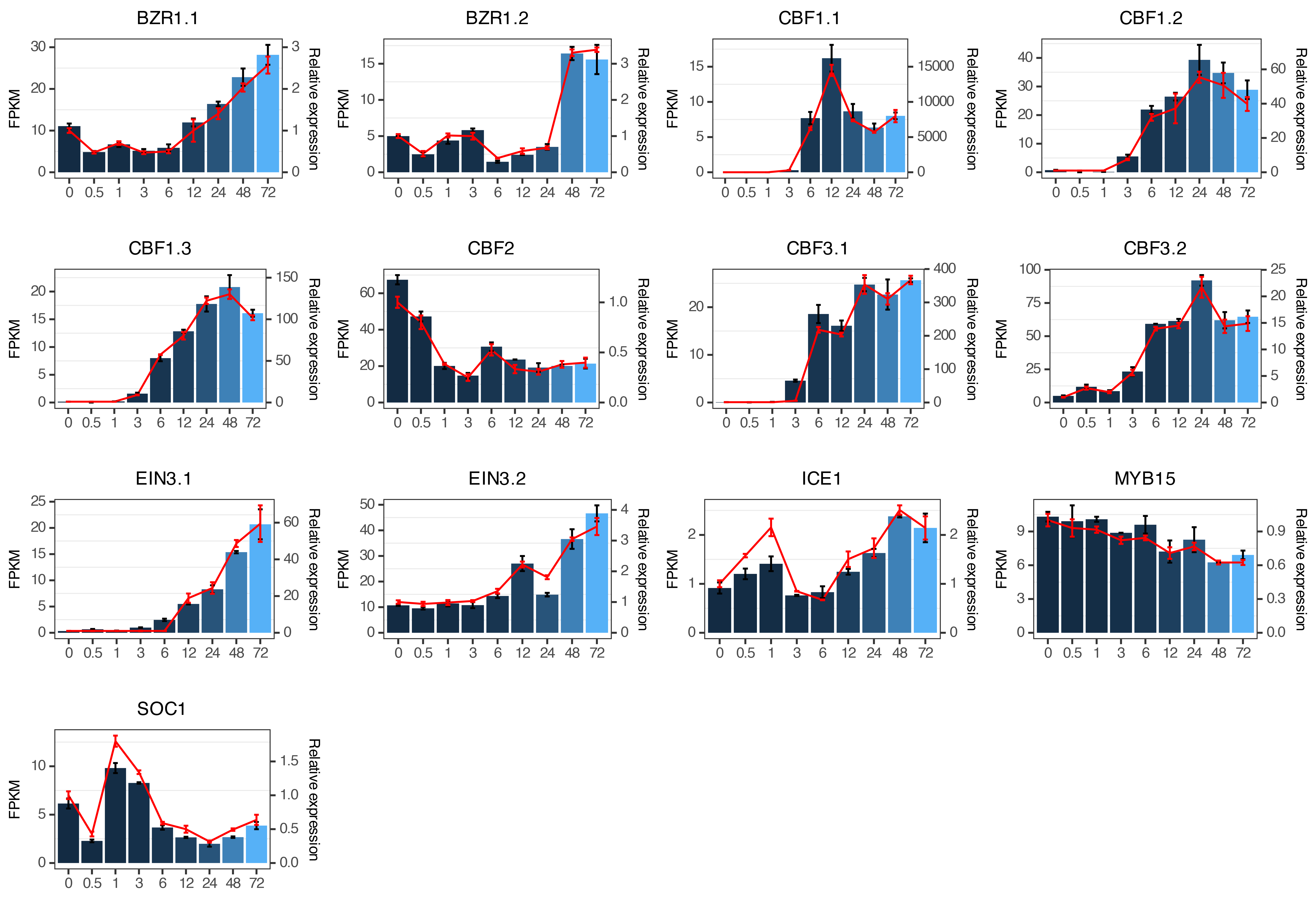

Supplement: Supplementary Figure 4 — Quantitative real-time PCR (qRT-PCR) validation the 13 ICE-CBF-COR genes of RNA-seq. FPKM values (left) and relative expression (right) of genes in different cold times. [file Image4.tif]
